# Evolutionary study of duplications of the miRNA machinery in aphids associated with striking rate acceleration and changes in expression profiles

**DOI:** 10.1186/1471-2148-12-216

**Published:** 2012-11-12

**Authors:** Benjamín Ortiz-Rivas, Stéphanie Jaubert-Possamai, Sylvie Tanguy, Jean-Pierre Gauthier, Denis Tagu, Rispe Claude

**Affiliations:** 1 INRA, UMR 1349 IGEPP, Le Rheu, 35653, France; 2 INRA, UMR1355, Institut Sophia Agrobiotech, Sophia Antipolis, 06903, France

**Keywords:** Aphids, miRNAs, Gene duplication, Neofunctionalization

## Abstract

**Background:**

The sequencing of the genome of the pea aphid *Acyrthosiphon pisum* revealed an unusual expansion of the miRNA machinery, with two *argonaute-1*, two *dicer-1* and four *pasha* gene copies. In this report, we have undertaken a deeper evolutionary analysis of the phylogenetic timing of these gene duplications and of the associated selective pressures by sequencing the two copies of *ago-1* and *dcr-1* in different aphid species of the subfamily Aphidinae. We have also carried out an analysis of the expression of both copies of *ago-1* and *dcr-1* by semi-quantitative PCR in different morphs of the pea aphid life cycle.

**Results:**

The analysis has shown that the duplication of *ago-1* occurred in an ancestor of the subfamily Aphidinae while the duplication of *dcr-1* appears to be more recent. Besides, it has confirmed a pattern of one conserved copy and one accelerated copy for both genes, and has revealed the action of positive selection on several regions of the fast-evolving *ago-1b*. On the other hand, the semi-quantitative PCR experiments have revealed a differential expression of these genes between the morphs of the parthenogenetic and the sexual phases of *Acyrthosiphon pisum*.

**Conclusions:**

The discovery of these gene duplications in the miRNA machinery of aphids opens new perspectives of research about the regulation of gene expression in these insects. Accelerated evolution, positive selection and differential expression affecting some of the copies of these genes suggests the possibility of a neofunctionalization of these duplicates, which might play a role in the display of the striking phenotypic plasticity of aphids.

## Background

Evolutionary novelty may brought by different processes, their different weights having been debated in the last years [1]. These processes include changes in protein sequence, regulatory changes and duplication [2], or combinations of all these factors [3]. The present study illustrates a possible new example of a combination of duplication, modulation of gene expression and changes in evolutionary rates, which may be of relevance to adaptation in a group of insects.

Small non-coding RNAs have been shown to play a fundamental role in the regulation of gene expression. These ~20-30 nucleotides long molecules have been described (e.g. endo- and exo-small interfering RNAs, micro RNAs, piwi associated RNAs…) and classified depending on their endogenous or exogenous origin, their mechanisms of biogenesis and function and their biological role. Regulation of gene expression by small non-coding RNAs is always carried out in association with a protein of the Argonaute family and is executed at multiple levels, ranging from chromatin modification to post-transcriptional control [4,5].

MicroRNAs (miRNAs) are small non-coding RNAs of ~20-24 nucleotides which derive from the transcription of endogenous genes by RNA pol II. In animals, they are first produced as long stem-loop hairpin primary miRNA precursors (pri-miRNA) which are cleaved in the nucleus by the RNase III enzyme Drosha with the help of the double stranded RNA binding protein Pasha (DGCR8 in mammals) [6]. The resulting pre-miRNAs are then translocated to the cytoplasm by Exportin-5, where they are subsequently cleaved by a second RNase III enzyme called Dicer in association with the double stranded RNA binding protein Loquacious (TRBP in mammals), yielding the mature miRNA in the form of an RNA duplex. Commonly, one of the strands of the duplex is degraded while the other is loaded into an effector complex named RISC (RNA-induced silencing complex) which has a protein of the Argonaute family as key component. The miRNA guides the RISC to a target messenger RNA (mRNA) by recognition of a complementary or nearly complementary sequence usually located in the 3’UTR of the target mRNA. MicroRNA-mRNA recognition leads to the degradation or the inhibition of the translation of the targeted mRNA [7-9].

Several instances of duplications have been reported for the machinery of small non-coding RNAs, in particular for the miRNA pathway in animals. Some of these duplications have been also linked to the neofunctionalization or subfunctionalization of one or both duplicate pairs. For example, only one Dicer is present in vertebrates and in the nematode *Caenorhabditis elegans*, which cleaves both miRNAs and small interfering RNAs (siRNAs), but two copies of this protein have been described in *Drosophila melanogaster* and other insects: Dicer-1, involved in the miRNA pathway, and Dicer-2, associated with the production of siRNAs [10]. Expansions of the Argonaute family have been more common, the most impressive example being the 27 members found in *C. elegans*[11]. The duplications in this nematode have involved subfunctionalization and neofunctionalization of the copies, with only two of the 27 copies associated with miRNAs. Other expansions of this family have also been observed in insects like the mosquitoes *Culex pipiens* and *Aedes aegypti*[12], or in the crustacean *Daphnia pulex*, with 9 copies described [13]. In *D. melanogaster* five Argonaute proteins exist, of which AGO-1 (mainly associated with miRNAs) and AGO-2 (mainly with siRNAs) are related to the RISC. On the contrary, all of the 4 Argonaute proteins described in vertebrates are able to load miRNAs [4,14].

The sequencing of the genome of the pea aphid *Acyrthosiphon pisum*[15] allowed the identification of homologues of the small non-coding RNAs machinery for the first time in a hemimetabolous insect. Their preliminary characterization revealed an unusual expansion of the machinery specific to piRNAs [16] and miRNAs, with the existence of four copies of Pasha (*Apipasha-1* to *Apipasha-4*), two copies of Dicer-1 (*Apidcr-1a* and *Apidcr-1b*), and two copies of Argonaute-1 (*Apiago-1a* and *Apiago-1b*) [17]. This is the first such general expansion of the miRNA machinery observed for a coelomate animal. In all other insects studied to date, only one copy of each of these genes specific to the miRNA pathway exists. Preliminary molecular evolutionary analyses suggested that the duplications of *ago-1* and *dcr-1* in aphids occurred roughly simultaneously, and that for each gene one of the duplicated copies was characterized by a higher evolutionary rate and relaxation of selection (*ago-1b* and *dcr-1b*), while the other was conserved and subject to strong purifying selection (*ago-1a* and *dcr-1a*). Furthermore, the ratio of non-synonymous to synonymous rates (dN/dS or ω) was well above 1 for *ago-1b*, which suggested the existence of positive selection driving the evolution of this gene, possibly towards a new function [17]. However, most of these preliminary analyses included only one aphid species, *A. pisum*, which might have given very rough estimates of the dates of duplication events and of the selective pressures acting on the different copies.

To further evaluate the biological implications of the expansion of the miRNA machinery in aphids, we have carried out an evolutionary and expression analysis of *dcr-1* and *ago-1* duplicated genes. For this, the two copies of *dcr-1* and *ago-1* were sequenced in several aphid species representing a gradient of evolutionary distance from *A. pisum* in order to better evaluate the phylogenetic timing of the duplications as well as the evolutionary pattern of nucleotide evolution and selective pressures affecting these genes.

The existence of different copies of the miRNA machinery raises the question of whether these duplicates could be recruited differently along the complex biological life-cycle of aphids, and if the different copies could play a role in the modulation of gene expression along this cycle. Aphids typically alternate between several generations of parthenogenetic females, which take place during spring and summer, and one annual generation of sexual reproduction at the end of summer, which produces overwintering eggs. They display a remarkable degree of polyphenism, with several morphs resulting from a same genotype during the parthenogenetic phase of their life cycle (e.g. winged/wingless individuals, viviparous/oviparous females …). As a first attempt to evaluate the functional implication of the miRNA machinery duplications, semi-quantitative RT-PCRs were carried out in the different reproductive morphs of the pea aphid and revealed different levels of gene expression in parthenogenetic and sexual aphids, suggesting a role for *ago-1b* and *dcr-1b* regulation in the sexual polyphenism of *A. pisum*. We discuss the data in the perspective that the duplication of *ago-1* and *dcr-1* might be related to the neofunctionalization of one of the copies of each of these genes.

## Results

### Phylogenetic distribution of the duplications of the miRNA machinery in aphids

We have investigated the duplication of *dcr-1* and *ago-1* in several aphid species from the subfamily Aphidinae. Two copies of *ago-1* were found in all of these species, belonging to two tribes, Aphidini and Macrosiphini (see Table 1). However, some regions of *ago-1b* could not be obtained in some species, due to unsuccessful PCR amplifications.

**Table 1:** **EMBL Accession numbers of genes from aphid species analysed in this study**^**a**^

	**Species**^**b**^	***ago-1a***			***ago-1b***			***dcr-1/dcr-1a***^**c**^	***dcr-1b***
		**Region 1**	**Region 2**	**Region 3**	**Region 1**	**Region 2**	**Region 3**		
**Macrosiphini**	*Acyrthosiphon pisum*	HE585884 HE585889	HE585923	HE585940	HE585898 HE585904	HE585918 HE585919	HE585950 HE585952	HE585973	HE585961 HE585966
	*Acyrthosiphon kondoi*	HE585892 HE585893	HE585926	HE585935	HE585905	HE585912	HE585953	HE585979 HE585980	HE585963
	*Acyrthosiphon svalbardicum*	HE585896	HE585927	HE585934	HE585906^e^	HE585913	HE585947	HE585968	HE585962
	*Sitobion avenae*	HE585902	HE585929 HE585930	HE585942 HE585943	HE585883	HE585914	HE585945	HE585978	
	*Aulacorthum solani*	HE585894 HE585895	HE585928	HE585941	HE585907^e^	HE585920	HE585944 HE585948	HE585976 HE585977	
	*Myzus persicae*	HE585886	HE585931	HE585946	HE585897	HE585921		HE585969 HE585970	
**Aphidini**	*Aphis gossypii*	HE585882	HE585910 HE585911	GW549708^d^		HE585981 HE585982^e^	HE585955	HE585959	
	*Rhopalosiphum padi*	HE585880 HE585881	HE585908 HE585909	HE585932 HE585933			HE585956	HE585957 HE585958	

^**a**^EMBL accession numbers are given for each copy and region of *ago-1* and for each copy of *dcr-1*. Two numbers are given for those species where two alleles were obtained. A blank in a cell means that the sequence for that gene/region in that particular species was not obtained. ^**b**^Aphid species were classified following Remaudière and Remaudière (1997). ^**c**^The gene was named *dcr-1a* in the *Acyrthosiphon* species, where a *dcr-1b* sequence was found, and *dcr-1* in the rest of species, where a second copy was not found. ^d^Sequence from an *Aphis gossypii* cDNA library (Webb, B.A. and Shelby, K.S., unpublished data). ^e^Sequences for which the complete targeted region could not be obtained.

**Figure 1:**
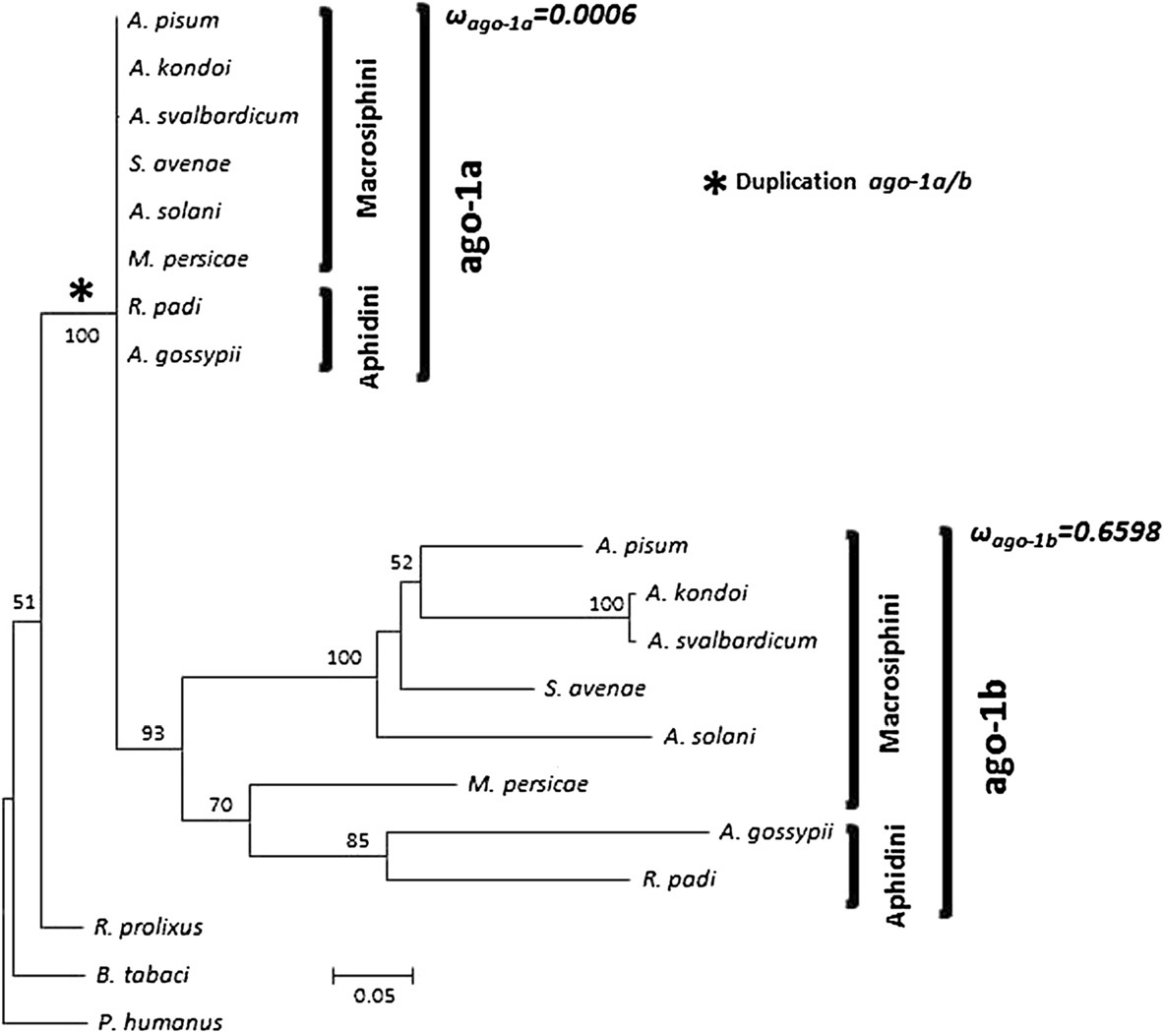
**Maximum likelihood tree inferred from the amino acid concatenated alignment of *ago-1 *sequences (model JTT+G+F).** The same topology was found using maximum parsimony and Bayesian inference, with only slight differences in the relationships inside the *ago-1b* group. For those species for which two alleles were obtained only one consensus sequence was included, since no correlation could be made between different alleles of the three different regions. An asterisk (*) marks the suggested moment of the duplication of *ago-1*. Bootstrap values are shown only when higher that 50%. For each copy of the gene, the value of the ratio of non-synonymous to synonymous rates (ω) resulting from the “two-ratio” model is shown (see Results). The value was calculated as the weighted mean among the ω values of the three coding nucleotide regions analyzed. A strong purifying selection characterizes the *ago-1a* sequences, contrasting with relaxed selection of *ago-1b*.

The separation of *ago-1a* and *ago-1b* copies was clear when amino acid sequences were used for the phylogenetic inference, for the three regions of the gene as well as for the concatenated alignment, and independently of the method used (Figure 1), while with nucleotide sequences the groups differed depending on the phylogenetic method. All amino acid sequences of *ago-1a* in the Aphidini+Macrosiphini were identical except for one amino acid substitution in Region 3 in *A. svalbardicum*. By contrast, *ago-1b* copies were much more variable, as revealed by long branches for all species.

**Figure 2:**
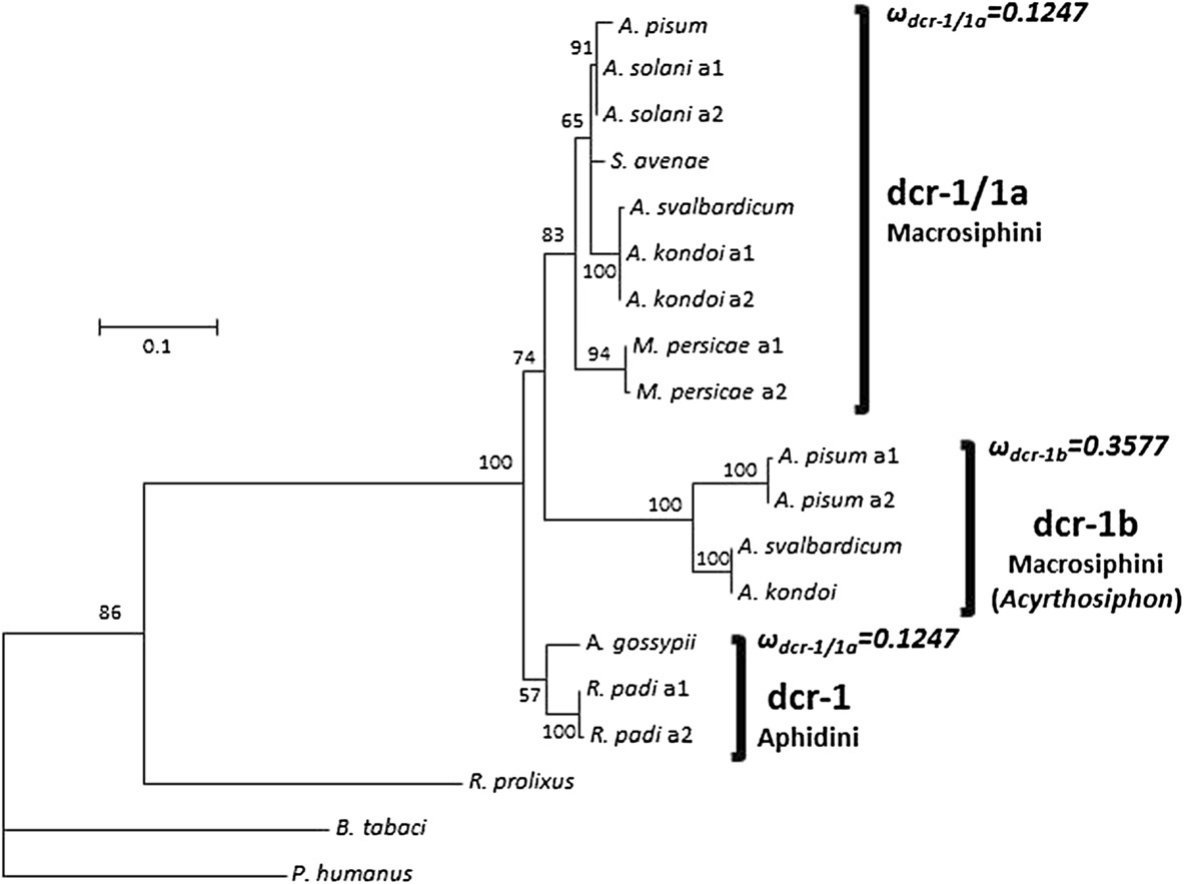
**Maximum likelihood tree inferred from the amino acid alignment of *dcr-1 *sequences.** The same topology was found with Bayesian inference and maximum parsimony except for the lack of monophyly of the Aphidini in the latter method. Bootstrap values are shown only when higher that 50%. a1: allele 1; a2: allele 2. For each copy of the gene, the value of the ratio of non-synonymous to synonymous rates (ω) resulting from the “two-ratio” model is shown (see Results), reflecting the difference in evolutionary pressures acting on the different copies, though less marked than for *ago-1*.

The sequencing of *dcr-1* yielded two different copies only in the three species of the genus *Acyrthosiphon*. Despite the use of up to 4 combinations of PCR primers, no partial *dcr-1b* sequence was obtained in the other species. The name of the gene was kept as *dcr-1* for those species where only one copy was sequenced, while the two copies found in the *Acyrthosiphon* species were named *dcr-1a* or *dcr-1b.* Identical trees were obtained with different methods and whether analyzing nucleotide or amino acid data. By contrast with results obtained for ago-1, the oldest nodes in the maximum likelihood tree obtained for dcr-1 were not supported by high bootstrap values, thus preventing an unambiguous determination of the phylogenetic timing of the duplication of this gene in aphids. We conducted SH and ELW [18,19] tests to compare among seven different phylogenetic hypotheses concerning the time of duplication of dcr-1. The seven hypotheses were constructed by independent permutation of each of the three oldest nodes in the dcr-1 ML tree (Figure 2; nodes supported by 57%, 74% and 83% bootstrap values). The ML tree was named as hypothesis 1. The seven hypotheses can be seen in Additional file 1: Figure S3. Hypotheses 2 and 3 placed either *R. padi* dcr-1 or *A. gossypii* dcr-1 sequences in a basal position, leaving the other one as sister of the rest of aphid sequences. Hypotheses 4 and 5 included *R. padi* and *A. gossypii* dcr-1 sequences in the dcr-1/1a group or the dcr-1b group, respectively. Hypotheses 6 and 7 altered the phylogenetic position of *M. persicae* dcr-1 sequences, either placing them as sister to the rest of Macrosiphini or included in the dcr-1b group.

None of the seven hypotheses were rejected by the SH test, while the ELW test only rejected hypotheses 6 and 7 (Table 2). The hypothesis 1 (Figure 2) suggests one gene duplication of dcr-1 that would have happened in the ancestor of the tribe Macrosiphini, after the split between Aphidini and Macrosiphini; in addition, the dcr1-b copy would have been lost secondarily in *M. persicae* or just could not be amplified in that species. The hypotheses 2 and 3 (not rejected in the tests) imply a complex scenario for the evolution of this gene, involving several duplications during the evolution of the subfamily Aphidinae. Both hypotheses 2 and 3 would also involve that one of the copies would have been lost in the Macrosiphini or simply not amplified in the PCR experiments. The hypotheses 4 and 5 (not rejected in the tests) imply a simpler scenario that would imply that the duplication of dcr-1 would have preceded the split between Aphidini and Macrosiphini, and that the sequences obtained for *R. padi* and *A. gossypii* would belong either to the dcr-1a (hypothesis 4) or the dcr-1b (hypothesis 5) copies –but this again implies a loss of or failure to amplify the other copy in several species. Finally, the hypotheses 6 and 7, which involve a change in the branching of the *M. persicae* dcr-1 sequences, were rejected by the ELW test, which gives strong support to the belonging of these sequences to the dcr-1/1a group. Taken together, these results do not allow making a clear statement about the phylogenetic timing of the duplication of dcr-1 in aphids, although scenarios of a duplication after the split between Aphidini and Macrosiphini are more parsimonious.

**Table 2:** **Results from ELW and SH tests performed on the set of seven different hypotheses concerning the phylogenetic timing of *dcr-1 *duplication in aphids**

**Hypothesis**	**Brief description of topology**		**ELW**	**SH**
		**δ**	**c**	**p-value**
1	Amino acid ML tree (Figure 2)	0.87	0.1665	0.762
2	*R. padi dcr-1* basal	0.87	0.1673	0.761
3	*A. gossypii dcr-1* basal	Best	0.3947	1.000
4	Aphidini *dcr-1* in *dcr-1/1a* group	1.73	0.1122	0.546
5	Aphidini *dcr-1* in *dcr-1b* group	1.73	0.1123	0.546
6	*M. persicae dcr-1* basal to Macrosiphini	10.03	0.0292*	0.111
7	*M. persicae dcr-1* in *dcr-1b* group	10.36	0.0177*	0.091

The complete set of topologies can be found in Additional file 1: Figure S3. **δ:** difference in -lnL from best topology as calculated by TREE-PUZZLE. **c:** confidence value (expected likelihood weigth). A * denotes topologies significantly out of the confidence set in the ELW test and topologies significantly worse than the ML tree in the SH test.

### Analyses of evolutionary pressures on sequences

**Figure 3:**
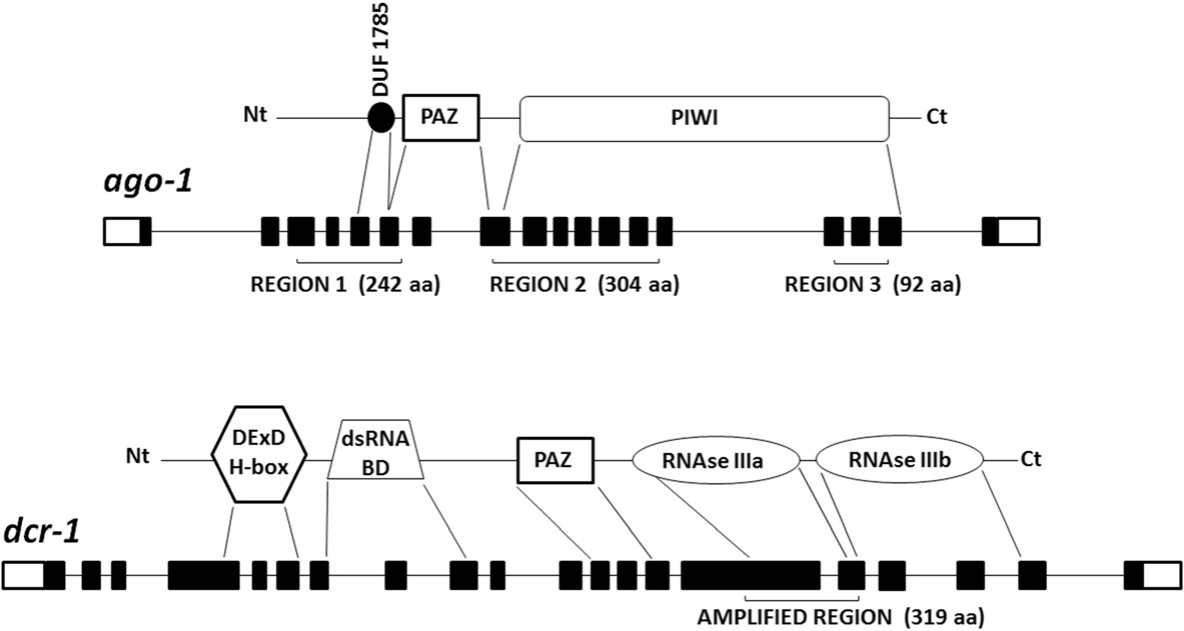
**Gene structure of *ago-1 *and *dcr-1 *in *Acyrthosiphon pisum*.** For each gene, both copies have a similar exon/intron structure. *Apiago-1a* and *Apiago-1b* have 18 exons, most of them packed in three different regions. The gene encodes a protein with an N-terminal domain of unknown function (DUF1785), a Piwi/Argonaute/Zwille domain (PAZ) and a C-terminal PIWI domain. *Apidcr-1a* and *Apidcr-1b* have 20 exons and encode a protein with an N-terminal DexD/H-box helicase domain, a dsRNA binding domain, a PAZ domain and two tandem C-terminal RNase III domains (RNase IIIa and IIIb). The RNase IIIa domain is truncated in *Apidcr-1b* by a deletion of 47 amino acids. The regions analyzed in this study are marked under the genes. Functional domains of the proteins are shown with approximate indication of the corresponding span in nucleotide sequence.

The best fit branch model for each region of *ago-1* and for *dcr-1* was the two-ratio model, with one ratio of non-synonymous to synonymous rates (ω=dN/dS) for the fast evolving copies (−*1b* copies) and a different ratio for the slow evolving copies (*ago-1a* or *dcr-1/1a*) copies (Table 3). This result is in agreement with the difference in branch lengths observed in the phylogenetic reconstructions (see above and Figures 1 and 2). However, selective pressures on each of the copies varied among regions and genes. Regions 1 and 2 of *ago-1* showed the minimal value of ω=0.0001 for *ago-1a* copies, due to lack of non-synonymous substitutions, so reflecting strong purifying selection acting on this copy. On the contrary, we found values of ω=0.8621 for Region 1 and ω=0.6236 for Region 2 for the *ago-1b* copies, suggesting highly relaxed selection. These two regions analyzed for *ago-1* encode an N-terminal domain of unknown function (DUF1785), a PAZ domain and the N-terminal portion of the PIWI domain of the protein (see Figure 3 for more details). The C-terminal portion of the PIWI domain is encoded in Region 3, where a similar pattern was found, but with a low value of ω=0.0039 for *ago-1a*, and a not so relaxed ω=0.2476 for the *ago-1b* copies. The difference between the slow- and fast-evolving copies of *dcr-1* was less pronounced, with ω=0.1247 for *dcr-1/1a* and ω=0.3577 for *dcr-1b*.

**Table 3:** **Summary of the analyses of selective pressures on *ago-1 *and *dcr-1 *with PAML**

	**Branch models**		**Site models**		**Branch-site models**	
***ago-1 *Region 1 (726 nt)**	one-ratio:**two-ratio**	P<0.0001				
	one-ratio:free-ratio	P<0.0001				
	**two-ratio**:free-ratio	P=0.6706	M7:**M8**	P<0.0001	MAof:**MAoe**	P=0.0285
	**ω**_***ago1a***_**=0.0001**	**ω**_***ago1b***_**=0.8621**	**4 sites with P>0.95**		**52 sites with P>0.95**	
***ago-1 *Region 2 (909 nt)**	one-ratio:**two-ratio**	P<0.0001				
	one-ratio:free-ratio	P<0.0001				
	**two-ratio**:free-ratio	P=0.0506	M7:**M8**	P<0.0001	MAof:**MAoe**	P=0.0218
	**ω**_***ago1a***_**=0.0001**	**ω**_***ago1b***_**=0.6236**	**5 sites with P>0.95**		**74 sites with P>0.95**	
***ago-1 *Region 3 (276 nt)**	one-ratio:**two-ratio**	P<0.0001				
	one-ratio:free-ratio	P<0.0001				
	**two-ratio**:free-ratio	P=0.4675	**M7**:M8	P=0.9802	**MAof**:MAoe	P=1
	**ω**_***ago1a***_**=0.0039**	**ω**_***ago1b***_**=0.2476**	**No positively selected sites**		**No positively selected sites**	
***dcr-1 *(957 nt)**	one-ratio:**two-ratio**	P=0.0002				
	one-ratio:free-ratio	P=0.0515				
	**two-ratio**:free-ratio	P=0.6424	**M7**:M8	P=0.4677	**MAof**:MAoe	P=1
	**ω**_***dcr-1/1a***_**=0.1247**	**ω**_***dcr-1b***_**=0.3577**	**No positively selected sites**		**No positively selected sites**	

For branch models, the best overall model is highlighted (two-ratio model for all the alignments) with its corresponding ω values. For site and branch-site models, the best model is highlighted as well as the number of positively selected sites when relevant. MAof: model MA with omega fixed to 1; MAoe: model MA with omega estimated. See text for further explanations about the models.

Consistent with this pattern, site models and branch-site models in which a class of positively selected codon positions is allowed (M8 and MA with omega estimated) were significantly better than neutral models (M7 and MA with omega fixed to 1) for Regions 1 and 2 of *ago-1*, but not for Region 3 of *ago-1* nor for *dcr-1*. Bayesian empirical Bayes estimates in model M8 showed 4 and 5 codon positions with a probability P>0.95 of ω being higher than 1 for Regions 1 and 2 respectively. The estimates from the branch-site model MA showed 52 and 74 codon positions with P>0.95 of ω>1 for the same regions. For both models the positively selected sites detected for Regions 1 and 2 did not aggregate in any particular domain.

### Gene conversion

GENECONV was used to search fragments in the alignments of *dcr-1* and *ago-1* that were unusually similar between pairs of sequences, a possible result from gene conversion. Some pairwise fragments were detected for two regions of *ago-1* and for *dcr-1* but always concerned two sequences of the same gene duplicate (two *-1a* copies or two *-1b* copies). On the contrary, no global fragments were significant for any of the three regions of *ago-1* or for *dcr-1*, thus showing no evidence for gene conversion occurring between the *-1a* and *-1b* copies of each of these genes.

### *Further duplication and pseudogenization of* dcr-1 *in the genus* Acyrthosiphon

The sequencing of *dcr-1a* and *dcr-1b* in the species *A. kondoi* and *A. svalbardicum* revealed a third copy of this gene, which was called *dcr-1c*. Furthermore, a deeper search on the *A. pisum* LSR1 genome led us to find also a third copy of *dcr-1* in this species, although partial and fragmented into two different positions of the currently available scaffolds of the genome (version 2 Assembly). Besides, the region of *dcr-1* analyzed in this study was not found in this third copy of *A. pisum*. The inclusion of these sequences in different phylogenetic trees showed that they correspond most likely to two independent further duplications of *dcr-1b* (see Additional file 2: Figures S1 and 3: Figure S2), one for *A. pisum* and another one for *A. kondoi* and *A. svalbardicum*. On the other hand, several features of these sequences, like the presence of frameshifts, stop codons and mutations in splice sites, strongly suggest that they have undergone a process of pseudogenization.

### *Alternative transcription of* dcr-1 *in aphids*

**Figure 4:**
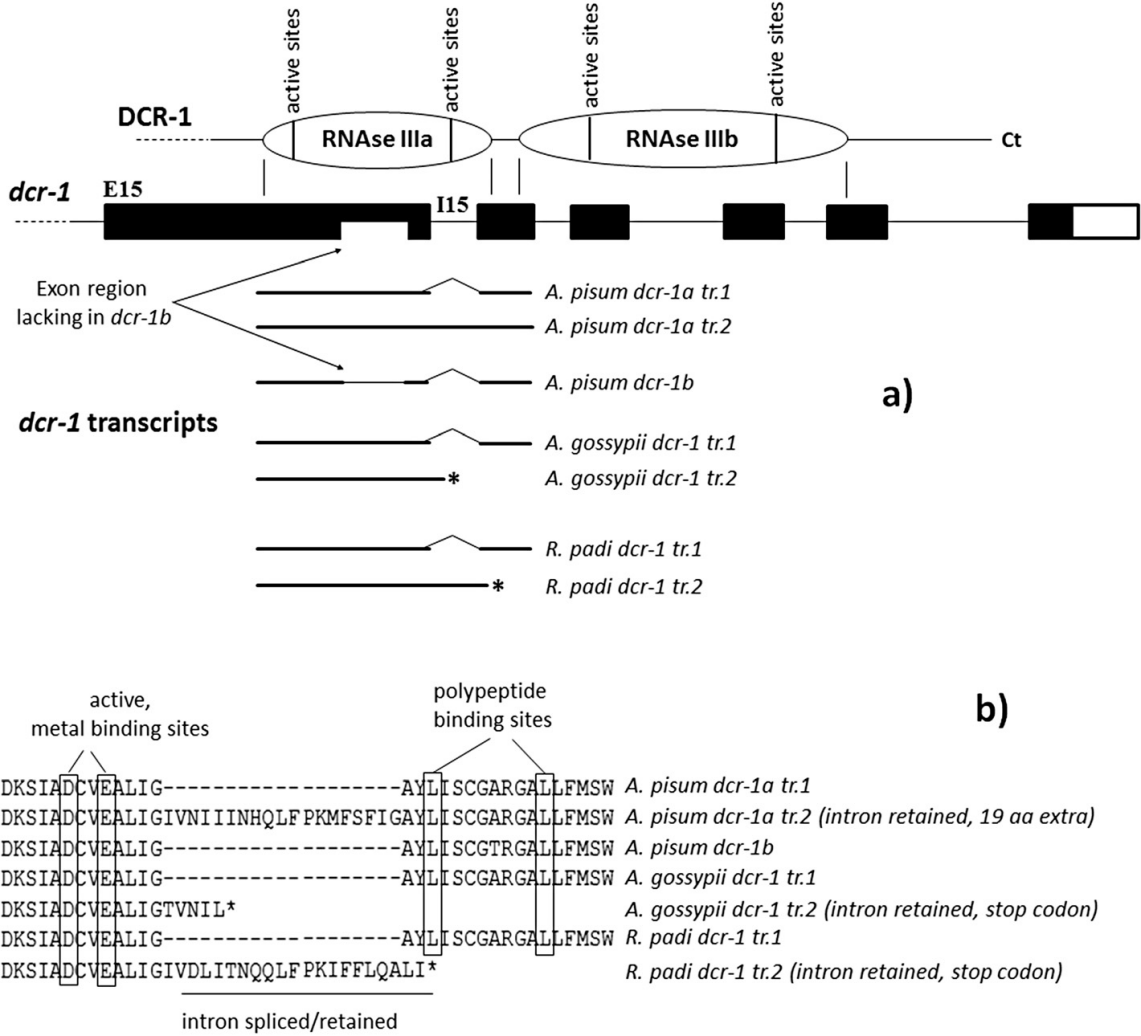
**Alternative transcription in *dcr-1/1a *in *A.pisum*, *A. gossypii *and *R.padi*. a**) Representation of the C-terminal region of DCR-1, the 3’ region of *dcr-1* and the transcripts sequenced for the three species. For each of them, two alternative transcripts were found for *dcr-1/1a*, one retaining intron 15 and another one splicing it. Only one transcript was found for *A. pisum dcr-1b*, with intron 15 spliced. *Apidcr-1b* lacks a portion of exon 15 as compared to *Apidcr-1a* which corresponds to a deletion of 47 aa in the middle of the RNase IIIa domain. **b**) Fragment of the alignment of the translated amino acid sequence of the transcripts, showing the region around intron 15. The retention of intron 15 results in an insertion of 19 aa in *A. pisum* DCR-1a. In *A. gossypii* and *R.padi* this retention results in premature stop codons (marked by an *) and the loss of the entire RNase IIIb domain of DCR-1.

The cloning of cDNA sequences from *A. pisum*, *A. gossypii* and *R. padi* revealed the existence of alternative transcription in *dcr-1/1a* (Figure 4). Two alternative transcripts were found for each of these three species, differing by a pattern of intron retention: in each species, intron 15 was retained in one of the transcripts but spliced in the other. In *A. pisum*, this intron is 57 nucleotides long, thus yielding a 19 amino acids longer protein, but in *A. gossypii* and *R. padi* this intron is 59 and 56 nucleotides long respectively, thus a not multiple of three. In these two latter species, intron retention introduces a frameshift and the appearance of premature stop codons. Intron 15 of *Apidcr-1* is located in the region of the gene that encodes the second set of functional sites of the RNase IIIa domain of the protein. The translation of the transcript retaining intron 15 in *A. pisum* would yield a separation of 19 aa between the active/metal binding sites and the polypeptide binding sites of these set. In *A. gossypii* and *R. padi*, this retention would entail the loss of these polypeptide binding sites and of the whole RNase IIIb domain of DCR-1 (see Figure 4).

### Expression profiles in the reproductive morphs

**Figure 5:**
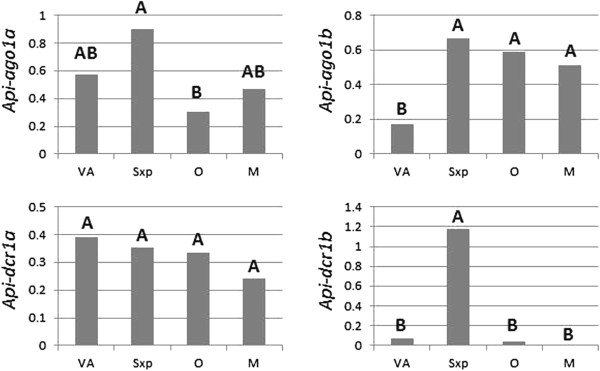
**Expression profiles of*Apiago-1a*,*Apiago-1b*,*Apidcr-1a*and*Apidcr-1b*in the four reproductive morphs of*A. pisum*LSR1.** Expression levels of the four genes were estimated by semi-quantitative RT-PCR in the four reproductive morphs: parthenogenetic virginoparae females (Vp), parthenogenetic sexuparae females (Sxp), sexual oviparae females (O) and sexual males (M). Expression was normalized with the gene encoding the ribosomal protein 7 (*Apirpl7*) as a reference gene [45]. The ratio between the expression of the amplified gene and of the *Apirpl7* reference gene was calculated to normalize for variations in experimental conditions, with three replicates. For each gene and morph, gene expression was measured from three batches of 10 adult aphids resulting from three independent biological experiments. The histograms show the averages of normalized expression levels, while letters indicate statistically significant differences among morphs (Duncan grouping in ANOVA).

Aphids typically alternate between several generations of parthenogenetic reproduction and one annual generation of sexual reproduction taking place at the end of summer in response to shortening of photoperiod [20]. Transcripts levels of *dcr-1a*, *dcr-1b*, *ago-1a* and *ago-1b* genes were measured by semi-quantitative RT-PCR in different reproductive morphs of the life cycle of the pea aphid *Acyrthosiphon pisum*: adult parthenogenetic virginoparae females (that contain parthenogenetic embryos in their ovaries), adult parthenogenetic sexuparae females (that contain sexual embryos in their ovaries), sexual oviparae females (that contain eggs in their ovaries), and males. The two *ago-1* gene copies and *dcr-1a* were expressed in all morphs (Figure 5). For *dcr-1a*, expression levels showed no statistically significant differences among morphs. By contrast, *ago-1a*, *ago-1b* and *dcr-1b* showed significant differences of expression according to the reproductive morph: *ago-1a*, was over-expressed in sexuparae and under-expressed in oviparae, and *ago-1b* was under-expressed in virginoparae while *dcr-1b* was over-expressed in sexuparae and not detected in males. These results suggest that the duplication of *ago-1* and *dcr-1* in the pea aphid is associated with specialization of transcription according to the reproductive morph.

## Discussion

The sequencing of the genome of the pea aphid *Acyrthosiphon pisum* indicated an unusual expansion of the small non coding RNA machinery specific to miRNAs [15,17]. The discovery of these gene duplications opens new perspectives of research about the regulation of gene expression by miRNAs in aphids, which might play a crucial role in the striking polyphenism displayed by these insects. Several scenarios may occur after gene duplications: non-functionalization, subfunctionalization and, more rarely, neofunctionalization [2]. Given that the miRNA machinery appears to be a fundamental mechanism in metazoa, with genes very conserved and as unique copies in most animal genomes known to date, it was particularly interesting to evaluate the different fates of these duplications. More precisely, we tried in this study to determine the age, evolutionary rates, and expression-specificities of the gene duplicates of *dicer-1* and *argonaute-1* described for *A. pisum*. The preliminary characterization of the expansion of the miRNA machinery in the pea aphid pointed out a strong divergence of *ago-1b* in *A. pisum*, a feature that suggested a change in function for AGO-1b protein in aphids [17].

The sequencing of *ago-1a* and *ago-1b* in different aphid species in the present study has revealed the presence of both copies of this gene in all the species analyzed, including the two tribes of the aphid subfamily Aphidinae: Aphidini and Macrosiphini. This result indicates that *ago-1* was duplicated in an ancestor of this subfamily (Figure 1). By contrast, our analysis provided a different estimation of the timing of duplication of *dcr-1*. We found a *dcr-1b* copy only in the three species of the genus *Acyrthosiphon*, which suggests a more recent duplication event. However due to the moderate bootstrap support values, we cannot presently propose a solid conclusion on the timing of this duplication. However, scenarios of a more recent duplication than found for ago-1 (scenarios in which the duplication of dcr-1 would only concern Macrosiphini) seem more parsimonious, as they imply less events of gene loss (or failure to amplify one copy in several species). Future genome sequencing projects for different aphids species will give the opportunity of estimating whether the duplication of *dcr-1* may have occurred earlier during aphid evolution.

Aphid genomes seem to have been affected by recurrent gene duplications through their evolution, with the pea aphid genome showing the highest number of gene families among the insect genomes sequenced to date [15,21]. In a previous study, we found similar pairwise synonymous distances between duplicates of genes of the miRNA machinery in the pea aphid, which suggested that the duplications of *ago-1* and *dcr-1* had occurred roughly simultaneously [17]. In contrast, in this study, with more phylogenetic evidence, we found that the duplication of *ago-1* probably preceded that of *dcr-1*. Indeed, we have also found that *dcr-1* has been further duplicated two independent times after the *dcr-1a*/*b* duplication in the genus *Acyrthosiphon*, although these new copies have most likely become pseudogenes.

The analysis of the patterns of non-synonymous and synonymous substitutions in the duplicates of *ago-1* and *dcr-1* in aphids has also provided, for each of the genes, a similar picture of a slow evolving copy subject to purifying selection (*ago-1a* and *dcr-1/1a*) and a fast evolving copy characterized by relaxed selection (*ago-1b* and *dcr-1b*). The difference among copies was much more pronounced for *ago-1* than for *dcr-1*. The evolution of *ago-1a* has clearly been driven by strong purifying selection, as detected by branch models in PAML, with extremely low values of ratios of non-synonymous to synonymous substitution rates (dN/dS or ω) and near-absence of replacements for the aphids of the subfamily Aphidinae. Such strong purifying selection implies sequence stability for this protein over a period of approximately 50–70 millions of years, the estimated age of the split between Aphidini and Macrosiphini [22]. Contrastingly, the *ago-1b* copies have been characterized by a strong acceleration of evolutionary rates, with different intensity in the three regions analyzed (Region 1: ω=0.8621; Region 2: ω=0.6236; Region 3: ω=0.2476). Branch models for the analysis of selective pressures estimate a dN/dS value that is averaged over the entire sequence analyzed. However, the gene sequence may contain both positions with very low or no variability due to purifying selection and positions in which change is promoted by positive selection. Site-models allow the detection of positive selection acting on specific positions of the sequence that might be unseen by branch models. Indeed, site and branch-site models led us to detect the signature of positive selection acting on Regions 1 and 2 of *ago-1b*. Branch-site models in particular showed that positive selection has played a role in the evolution of the *ago-1b* copies, with many codon positions with dN/dS ratios significantly higher than 1 (54 and 74 in Regions 1 and 2 respectively, which represent 22% and 24% of the codons of each region).

The difference of rates between the two copies of *dcr-1* was less pronounced than for the two copies of *ago-1*. The branch model of best fit estimated that purifying selection has characterized the *dcr-1a* copy of the *Acyrthosiphon* spp and the unique *dcr-1* copy in species where the duplication was not found, (ω=0.1247), although the sequence was less conserved than the slow-evolving *ago-1a*. By contrast, the selective pressures on *dcr-1b* appeared to be more relaxed (ω=0.3577) and similar to the value found for Region 3 in *ago-1b*. As for Region 3 of *ago-1b*, no sign of positive selection was detected in *dcr-1b*.

We finally evaluated differences of expression of the duplicated copies among different reproductive morphs characteristic of the aphid life-cycle, which alternates sexual and asexual reproduction. The display of such polyphenism must involve a finely tuned mechanism of regulation of gene expression from their gene families-rich genomes, in which miRNAs could play a key role, along with other phenomenon like alternative transcription. In this report, we have obtained a first insight on the functional characterization of the gene duplicates of *ago-1* and *dcr-1* by means of semiquantitative PCRs carried out on four morphs of the life cycle of the pea aphid. Our results have shown a differential regulation of the expression of *ago-1a*, *ago-1b* and *dcr-1b* among the morphs. The most striking pattern is that of *dcr-1b*, which seems to be only expressed in the sexupara, the parthenogenetic female that gives birth to the sexual morphs. Although no signature of positive selection could be detected for *dcr-1b*, the structure of its sequence gives an interesting clue supporting a possible change in function. The three species of *Acyrthosiphon* where *dcr-1b* was sequenced share a deletion of 47 amino acids in the protein sequence with respect to *dcr-1a*, which is located inside the first RNase III domain. This deletion does not affect any of the active sites, but has probably changed the distance between them inside the RNAse IIIa domain, as well as the distance between the RNAse IIIa and RNase IIIb domains. Interestingly, the two RNase III domains in Drosha and Dicer seem to interact with each other to make an intramolecular dimer with the two catalytic sites located close to each other, and the distance between them seems to determine the length of the small RNA produced after cleavage [14,23]. It is tempting to hypothesize that the deletion in DCR-1b, which has been evolutionarily conserved in the genus *Acyrthosiphon*, may have determined a change in the distance between catalytic centers and consequently in its function, leading to the production of small non coding RNAs of different length. All active sites in the two RNase III domains of DCR-1 have remained conserved in the two copies of the protein found in aphids and also in the three outgroup species except for two changes. By contrast, the region of the RNase IIIa domain that contains the deletion in DCR-1b is highly variable and shows other deletions in the outgroup species, which could affect the regulation of gene expression by small non coding RNAs. Consistent with this hypothesis, the alternative transcription described for *dcr-1a* in *A. pisum* would yield two translated DCR-1a proteins differing by the absence or presence of 19 aa located inside the second set of functional sites of the first RNase III domain of the protein, which might also alter the length between the catalytic centers and allow the production of small non coding RNAs of different length. The same alternative transcription observed in *A. gossypii* and *R. padi* entails the loss of the RNase IIIb domain in one of the alternative transcripts, which probably leads to an non-functional protein, given that the interaction of both RNase III domains seem to be needed for cleavage.

Future experiments will allow to study whether the gene duplications of the miRNA machinery play a primary role in the sexual/asexual polyphenism in aphids. It is important to note that cyclical parthenogenesis is an ancient and ubiquitous trait in these insects, and that most of its subfamilies display this same reproductive polyphenism with only one copy of DCR-1. This means that the duplication of the miRNA machinery genes was not essential for settling the sexual/asexual alternation of reproductive modes that define the aphid life-cycle. But the different copies might have evolved differently and specific roles at different steps of the life-cycle. The research on the implications of the miRNA machinery in the aphid polyphenism will benefit not only from comparative genomics analysis that will allow knowing precisely the phylogenetic distribution of its gene duplications but also comparative functional studies between aphids having and not having the gene duplicates. The strong purifying selection acting on *ago-1a* and *dcr-1a* suggests that these proteins probably have retained their function in the miRNA machinery. On the other side, both relaxed and positive selection acting on DCR-1b and AGO-1b might have led these copies to evolve a new function. Further studies will be needed to explore their potential new role in the miRNA system and at the different steps of the reproductive cycle of these organisms.

## Conclusions

The sequencing of the two gene duplicates of *argonaute-1* and *dicer-1* in several aphid species has allowed us to better estimate the phylogenetic timing and the evolutionary pressures that have affected the duplication of the miRNA machinery in these insects. This study has revealed that the duplication of *argonaute-1* occurred in the ancestor of the aphid subfamily Aphidinae, while the duplication of *dicer-1* seems to have occurred in the ancestor of the aphid tribe Macrosiphini. A common pattern for both genes has been shown with one fast-evolving copy (*ago-1b* and *dcr-1b*) and one slow-evolving copy (*ago-1a* and *dcr-1/1a*). The acceleration of evolutionary rates in *ago-1b* and *dcr-1b* and the existence of positive selection acting on *ago-1b* suggests the possibility of a neofunctionalization for these copies which might have interesting implications on the regulation of gene expression by miRNAs in aphids. Besides, semi-quantitative PCRs have revealed a differential expression of *ago-1b* and *dcr-1b* among the different morphs of the parthenogenetic and sexual phases of the pea aphid life cycle, thus suggesting their implication in the striking polyphenism displayed by this group of insects.

## Methods

### Aphid species analyzed

The aphid species used in this study (Table 1) belong to the subfamily Aphidinae (Hemiptera: Aphididae) and were chosen to cover a gradient of evolutionary distance from the pea aphid *A. pisum*. These include four species from the Macrosiphini tribe (including two other species of the genus *Acyrthosiphon*) and two species from the tribe Aphidini [24]. Sequences for outgroup species were retrieved from public databases, including two other Hemiptera species (*Bemisia tabaci* and *Rhodnius prolixus*) and a representative of the order Phthiraptera (*Pediculus humanus*).

### *PCR amplification of* ago-1 *and* dcr-1 *gene duplicates*

For *ago-1*, three different regions were chosen for the analyses, covering overall ~72% of the coding sequence. These regions were chosen to maximize the total coding length of the sequences obtained. Each region comprised a group of exons separated by short introns in both copies of *ago-1* in *A. pisum* (Figure 3), and we expected this pattern to be conserved in the different species. The regions were named Region 1 (exons 3 to 6), Region 2 (exons 8 to 14) and Region 3 (exons 15 to 17) and included several predicted functional domains of the protein. For *dcr-1*, a single sequence spanning exons 15 and 16 was analyzed.

Genomic DNA was extracted from a single individual or from several individuals of a same clone following a salting-out protocol [25]. In order to obtain transcript data to support predicted exon/intron boundaries, and also to amplify some regions not obtained from genomic DNA, RNA was extracted from whole body samples of *A. pisum* LSR1, *A. gossypii* and *R. padi*, with subsequent RT protocol carried out with Superscript III (Invitrogen).

Two alternative PCR approaches were carried out for the amplification of *ago-1* and *dcr-1* sequences: one using degenerate primers intended to amplify simultaneously both copies of the genes in a same reaction and another one using specific primers of copy *-1a* or *-1b*. PCR primers were based on the available sequence of *A. pisum* (primer sequences in Additional file 4: Table S1). In some instances, the initial primers did not allow the amplification of the targeted region so a smaller fragment was obtained by designing internal primers. A typical PCR reaction consisted in 94°C 3’, 10x(94°C 30”, 60°C decreasing to 50°C 60”, 72°C 60-120”), 30x(94°C 30”, 50°C 60”, 72°C 60-120”), 72°C 7’, 4°C hold, and was carried out with GoTaq system (Promega). Each amplified fragment was cloned using the Strataclone Package (Stratagene) following manufacturer’s instructions. For each sequence to be used in further analyses three or more clones were sequenced in order to detect *Taq* errors, deducing the final sequence as the consensus of the clones.

### Phylogenetic analyses

Chromatograms were analyzed and assembled using the Staden Package 2.0b [26]. Nucleotide sequences were aligned with Clustal X 2.0.9 [27] and corresponding amino acid alignments were obtained with MEGA 4.0.2 [28].

Phylogenetic trees were inferred from nucleotide and amino acid alignments by maximum likelihood, maximum parsimony and Bayesian inference. The analyses of *ago-1* were done separately for each of the three regions as well as for concatenated alignments. The phylogenetic analyses on nucleotide sequences were only carried out on coding regions. The model of sequence evolution for maximum likelihood analyses was chosen using jModeltest [29] for nucleotides and ProtTest [30,31] for amino acids. Maximum likelihood reconstructions were obtained with PhyML [32] using the NNi algorithm. PAUP* 4.0b10 [33] was chosen for maximum parsimony analyses with TBR branch swapping and 5000 repetitions of random sequence addition. Statistical support to nodes was evaluated for maximum likelihood and maximum parsimony by the bootstrap method [34] with 200 and 1000 pseudorreplicates respectively. The Bayesian inference of phylogeny was carried out as implemented in MrBayes 3.1 [35,36]. Two parallel runs, each one consisting of three cold and one heated chains were set. 10^6^ and 5 × 10^5^ generations for nucleotides and amino acids respectively were enough for reaching convergence between the runs, which was checked using Tracer v1.5 [37]. A burn-in fraction of the initial 25% generations was eliminated and posterior probabilities of trees were obtained by sampling every 100^th^ generation afterwards.

### Comparison of alternative hypotheses for dcr-1 duplication

SH tests [1] and ELW tests [2] were implemented in TREE-PUZZLE to compare among alternative scenarios concerning the phylogenetic timing of the duplication of dcr-1 in aphids. Seven different hypotheses were simultaneously tested in each test. The seven hypotheses were constructed by permutation of the groups branched to the oldest nodes in the maximum likelihood tree obtained from the amino acid alignment of dcr-1.

### Analyses of evolutionary pressures on sequences

The nature and distribution of the selective pressures acting on the gene duplicates of *ago-1* and *dcr-1* was evaluated using PAML 4.4 [38] on nucleotide coding regions. The analyses were carried out separately for each region of *ago-1* and for *dcr-1*, and outgroup sequences were excluded. Branch models were implemented to test the hypothesis of different ratios of non-synonymous to synonymous substitution rates (ω=dN/dS) acting on the *-1a* and *-1b* copies of these genes. Likelihood ratio tests (LRTs) were used to compare among: i) a “one-ratio” model that assumed no difference in the ratio across the phylogeny, ii) a “two-ratio” model that fitted one ratio for the fast evolving copies (−*1b* copies) and a different ratio for the slow evolving copies, and iii) a “free-ratio” model, that assumed the existence of a different ratio for each branch of the tree [39,40]. In the “two-ratio” model for *ago-1*, the *ago-1b* sequences were set as fast evolving copies and the *ago-1a* sequences as slow evolving copies. For *dcr-1*, the *dcr-1b* sequences were set as fast evolving copies and the rest of aphid *dcr-1* sequences as slow evolving copies (including the *dcr-1a* copies of *Acyrthosiphon*). Site-models were also used for the search of positively selected codon positions in the alignments. Two models were compared by an LRT test: model M7, assuming no positively selected sites in the alignment, and model M8, which allows for their existence [40,41]. The search for positively selected sites with these site models was made only for the *-1b* copies of the alignments. Finally, branch-site models were also implemented, comparing by an LRT the null model MA with ω fixed to 1 and the alternative model MA with ω estimated [42,43]. For branch-site models, both copies were included, labeling the clade of *-1b* copies as foreground branches, where positively selected sites are analyzed. All models were implemented allowing the four nucleotide frequencies to vary among codon positions (model F3X4), which gave significantly better likelihood values than not allowing variation (model F1X4).

### Test of gene conversion

The existence of gene conversion, particularly between the *-1a* and *-1b* copies of each of the genes, was evaluated by GENECONV [44]. The alignments used included all aphid sequences, with only one allele per species and no partial sequences. Each region of *ago-1* was analyzed separately. The program was run allowing the existence of mismatches in the fragments susceptible of having experienced gene conversion.

### Gene expression profiling in different reproductive morphs

The transcription of *dcr-1a*, *dcr-1b*, *ago-1a*, and *ago-1b* genes was measured by semi-quantitative reverse transcription-polymerase chain reaction (RT-PCR) in four reproductive morphs (all adult) of *Acyrthosiphon pisum* clone LSR1: parthenogenetic females (virginoparae), mothers of oviparae (sexuparae), sexual females (oviparae) and males. The gene encoding ribosomal protein 7 (*rpl7*) was used as a reference gene [45] to normalize *dcr-1* and *ago-1* expression. For each morph, three experimental replicates were done, consisting each of batches of 10 individuals which were frozen in liquid nitrogen 48 h after adult moult and used for RNA extraction. The concentration and quality of the extracted RNA was estimated with a NanoDrop (Thermo Scientific). First strand cDNAs were produced from 500 ng total RNA using the SuperscriptIII reverse transcriptase (Invitrogen) and random nonamers (Promega) following the supplier’s instructions. DNA contamination was removed by treating RNA extraction products with RNase-free DNAse (Promega). As a negative control, RT-PCR experiments were performed on total RNA without SuperscriptIII reverse transcriptase. The PCR program consisted of an initial step of 4 minutes (min) at 94°C, then multiple cycles composed of 2 min at 94°C, 30 seconds (sec) at the annealing temperature and 1 min 30 sec at 72°C, and a final elongation step of 5 min at 72°C. The appropriate number of cycles corresponding to the exponential range was defined for every gene in order to quantify the amplification products. Additional file 5: Table S2 lists the sequences of PCR primers, the corresponding annealing temperatures and the appropriate number of cycles used to measure expression level of the different *dcr-1* and *ago-1* genes. Images of the RT-PCR Sybr-safe (Invitrogen) stained agarose gels were acquired with a G:BOX Imager (Syngene) and quantification of the bands was performed using Image-J (http://rsbweb.nih.gov/ij/). For each batch of each reproductive morph, the ratio between the band intensity of the amplified gene and *rpl7* reference gene was calculated to normalize for initial variations in sample concentration. The significance of variation of expression level (p-value >0.05) was tested by analysis of variance (ANOVA) with un-transformed data.

## Competing interests

The authors declare that they have no competing interests.

## Authors’ contributions

BOR, SJP, DT and CR were in charge of the conception and design of the study. BOR gathered the sequence data for the phylogenetic and evolutionary analyses, which were carried out by BOR and CR. ST made the semi-quantitative PCR experiments, which were analyzed by SJP and JPG. The writing of the manuscript was done by BOR, SJP and CR, with revision by DT. All authors read and approved the final manuscript.

## Supplementary Material

Additional file 1 **Figure S3.** Test of alternative hypotheses for the phylogeny of *dcr-1* in aphids. Click here for file

Additional file 2 **Figure S1.** Further duplication of *dcr-1* in *Acyrthosiphon kondoi* and *Acyrthosiphon svalbardicum.* Click here for file

Additional file 3 **Figure S2.** Further duplication of *dcr-1* in *Acyrthosiphon pisum*LSR1. Click here for file

Additional file 4 **Table S1.** PCR primers used in this study, ordered in relative positions from 5’ to 3’ of the corresponding gene/region. Click here for file

Additional file 5 **Table S2.** PCR primers used for semiquantitative RT-PCRs. Click here for file
