# Supplementary figures and images for: Evolutionary study of duplications of the miRNA machinery in aphids associated with striking rate acceleration and changes in expression profiles
Source: BMC Evol Biol. 2012 Nov 12;12:216. doi: 10.1186/1471-2148-12-216 (PMC3536612; doi:10.1186/1471-2148-12-216)

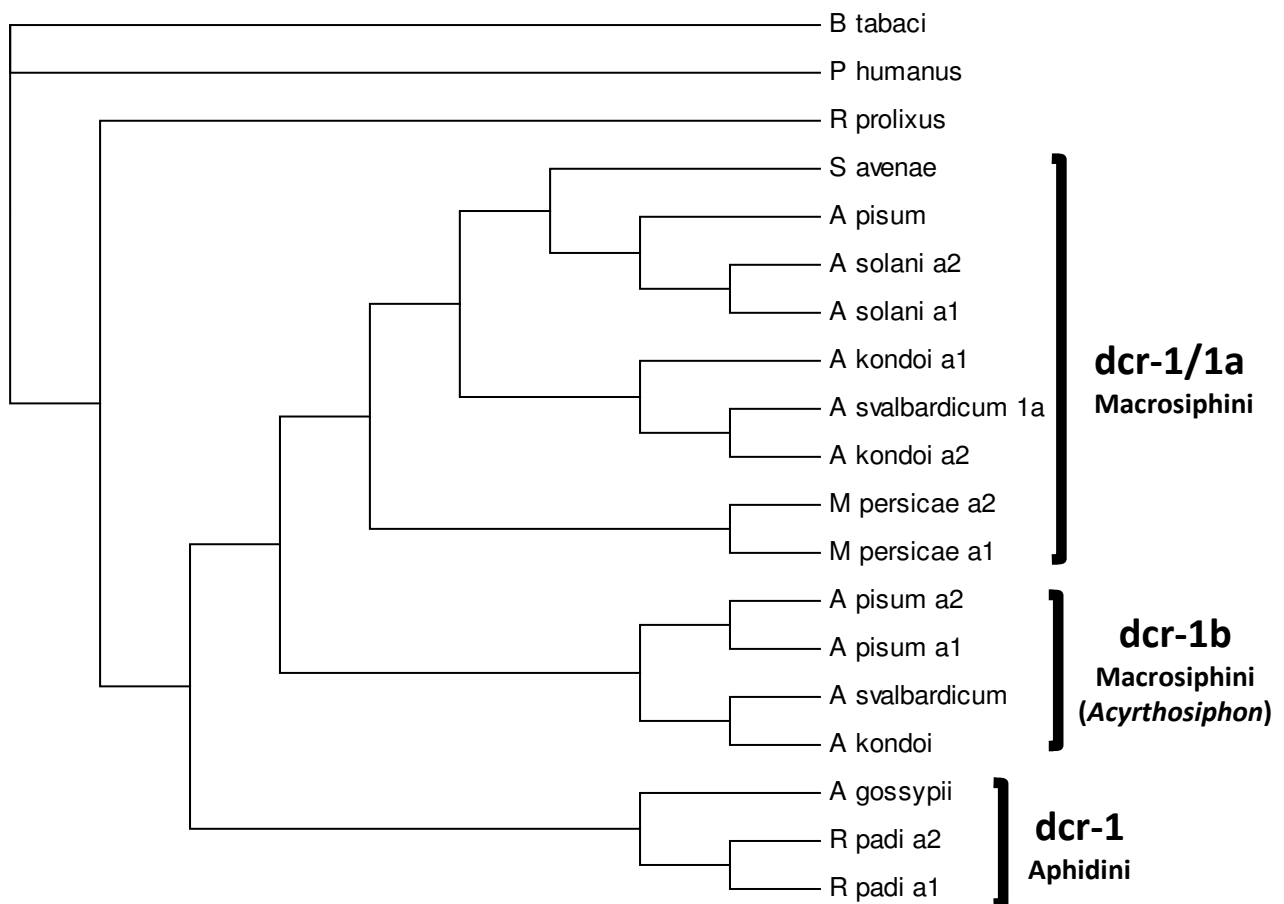

## Hypothesis 1

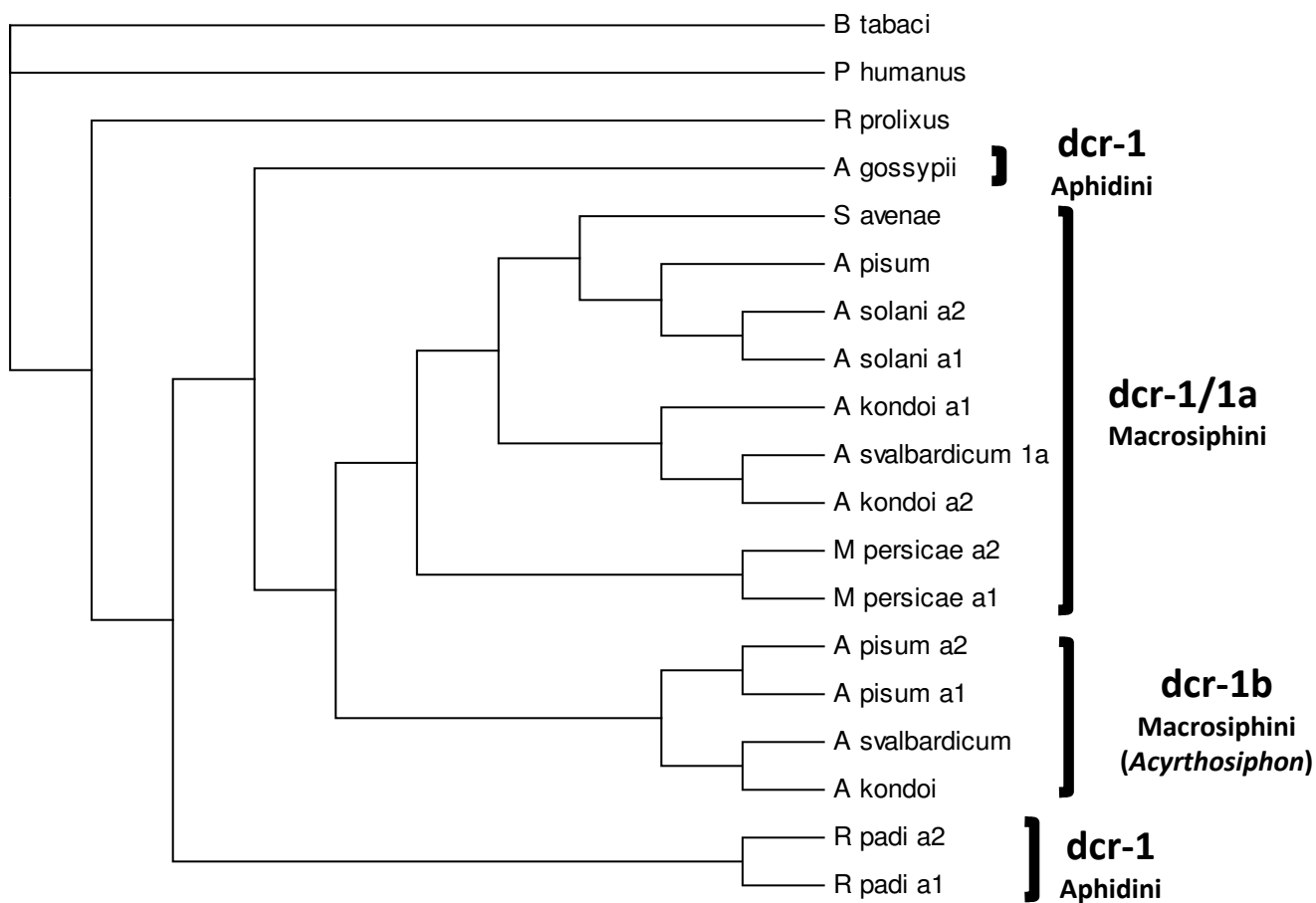

## Hypothesis 2

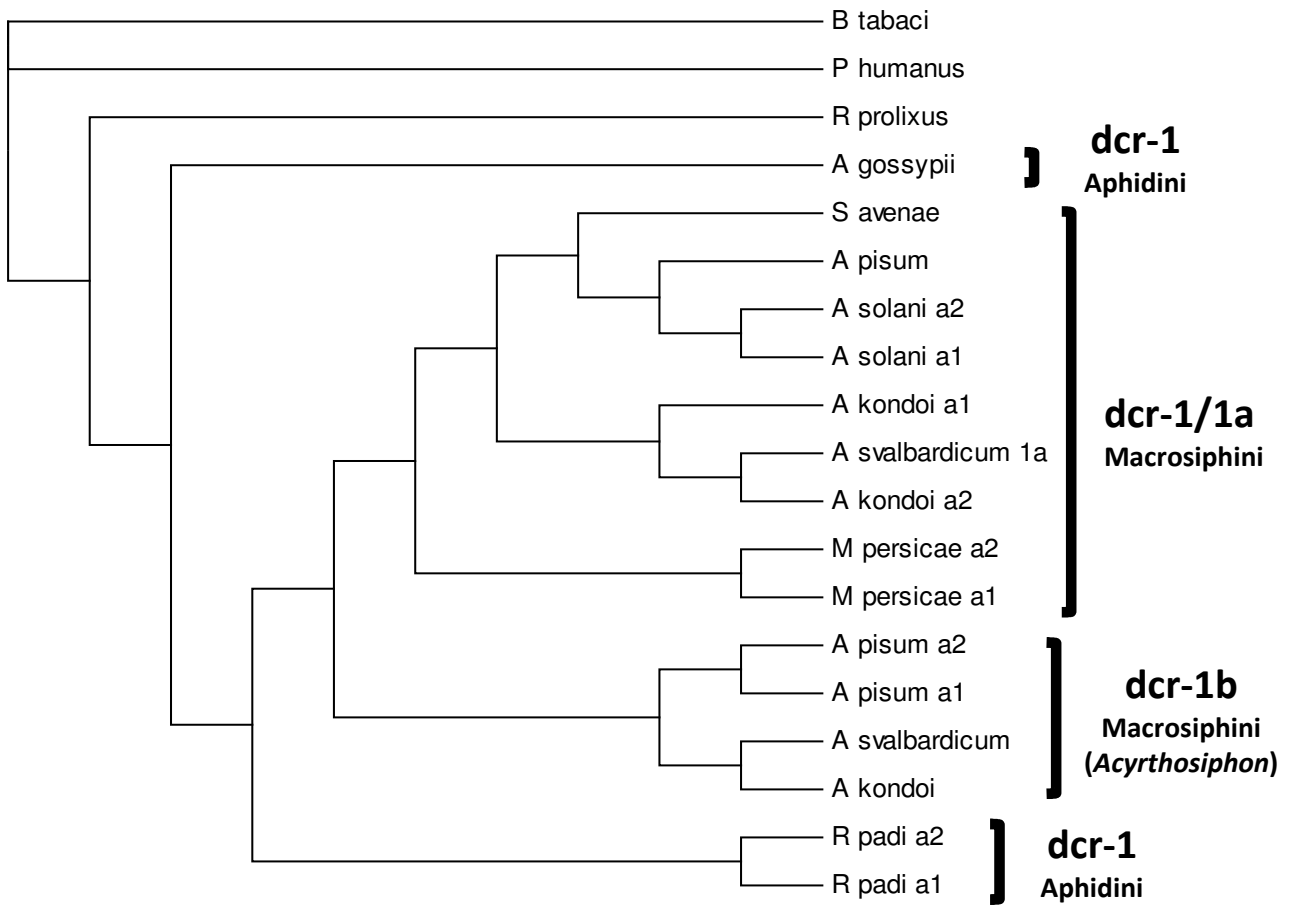

## Hypothesis 3

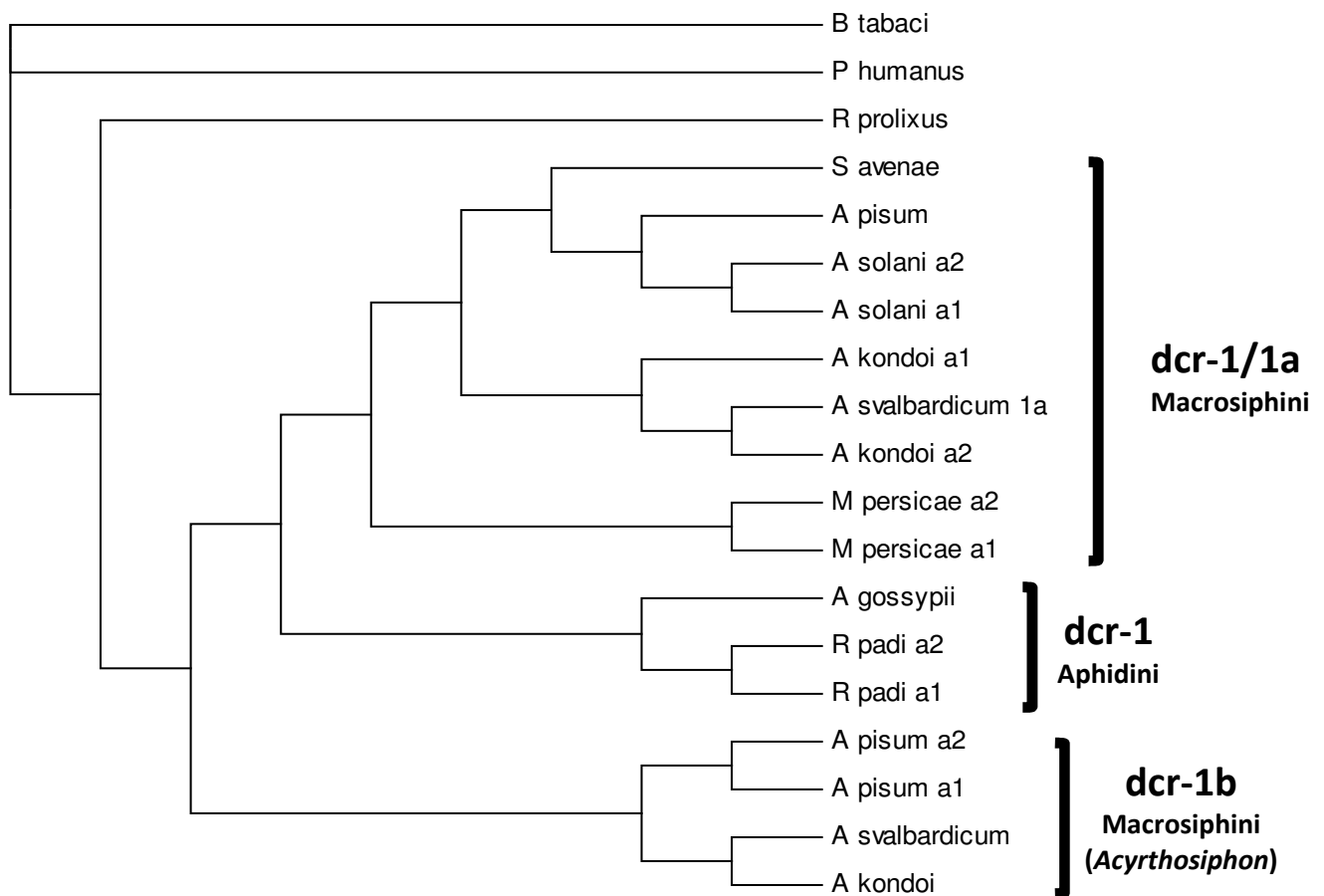

## Hypothesis 4

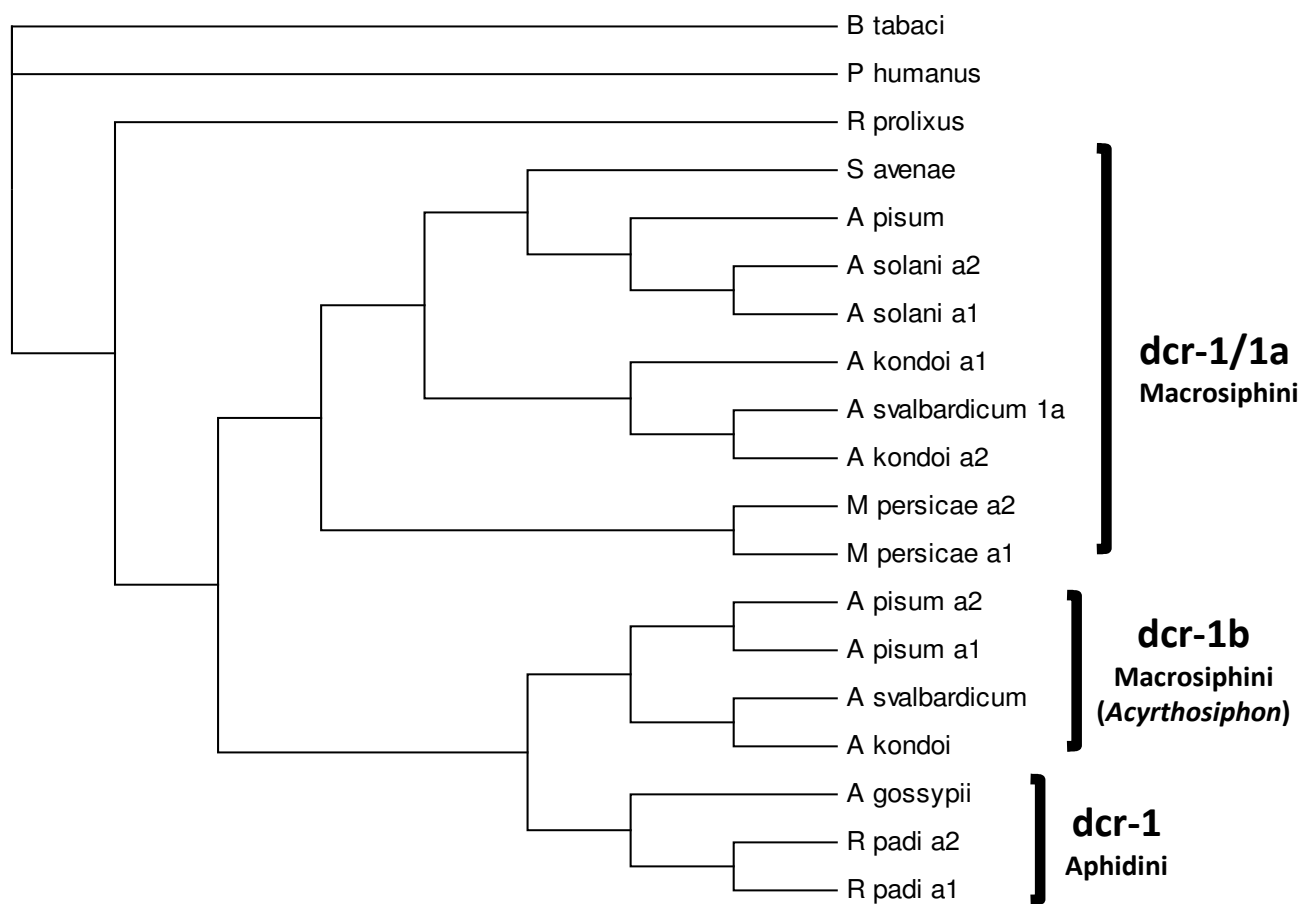

## Hypothesis 5

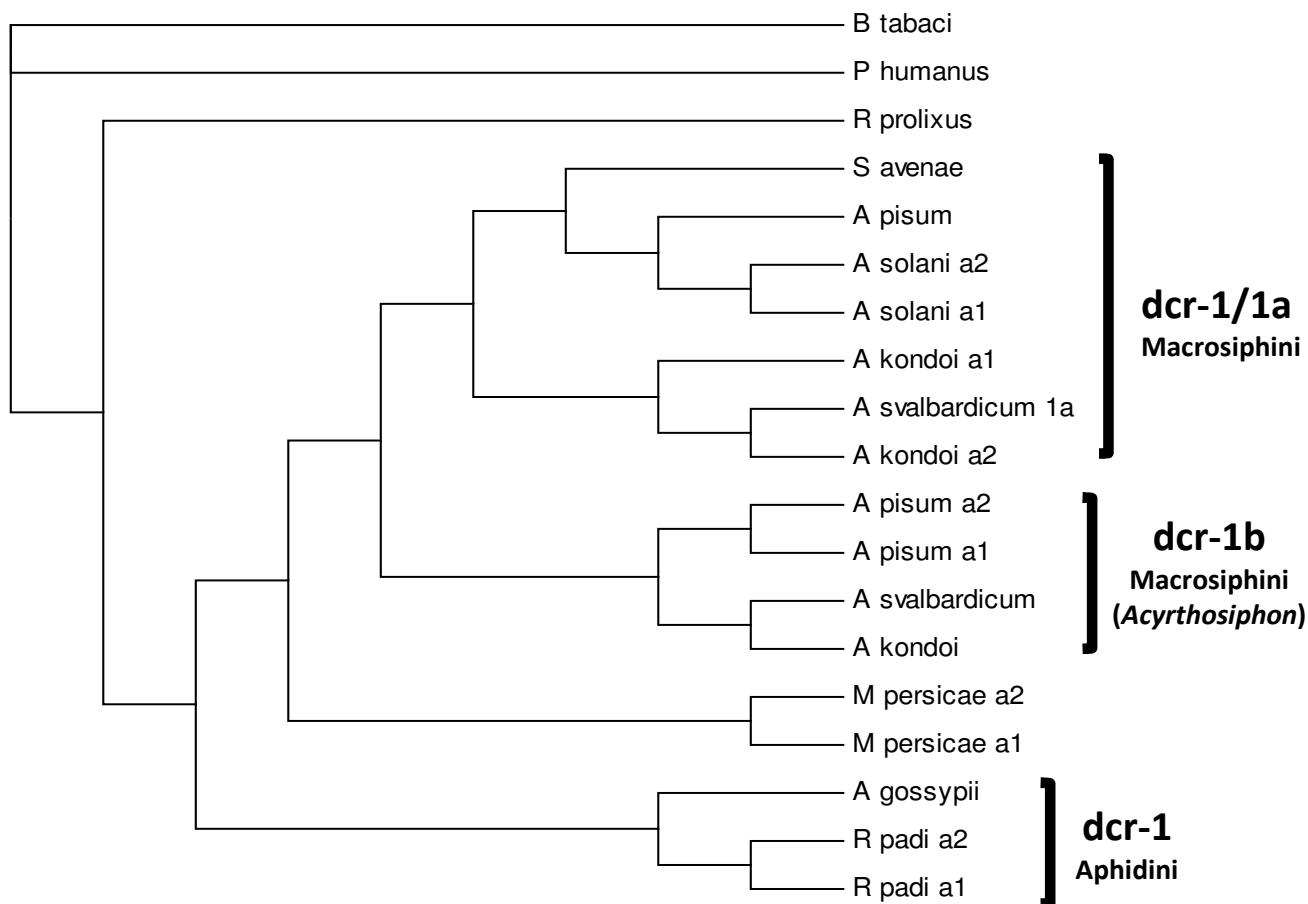

## Hypothesis 6

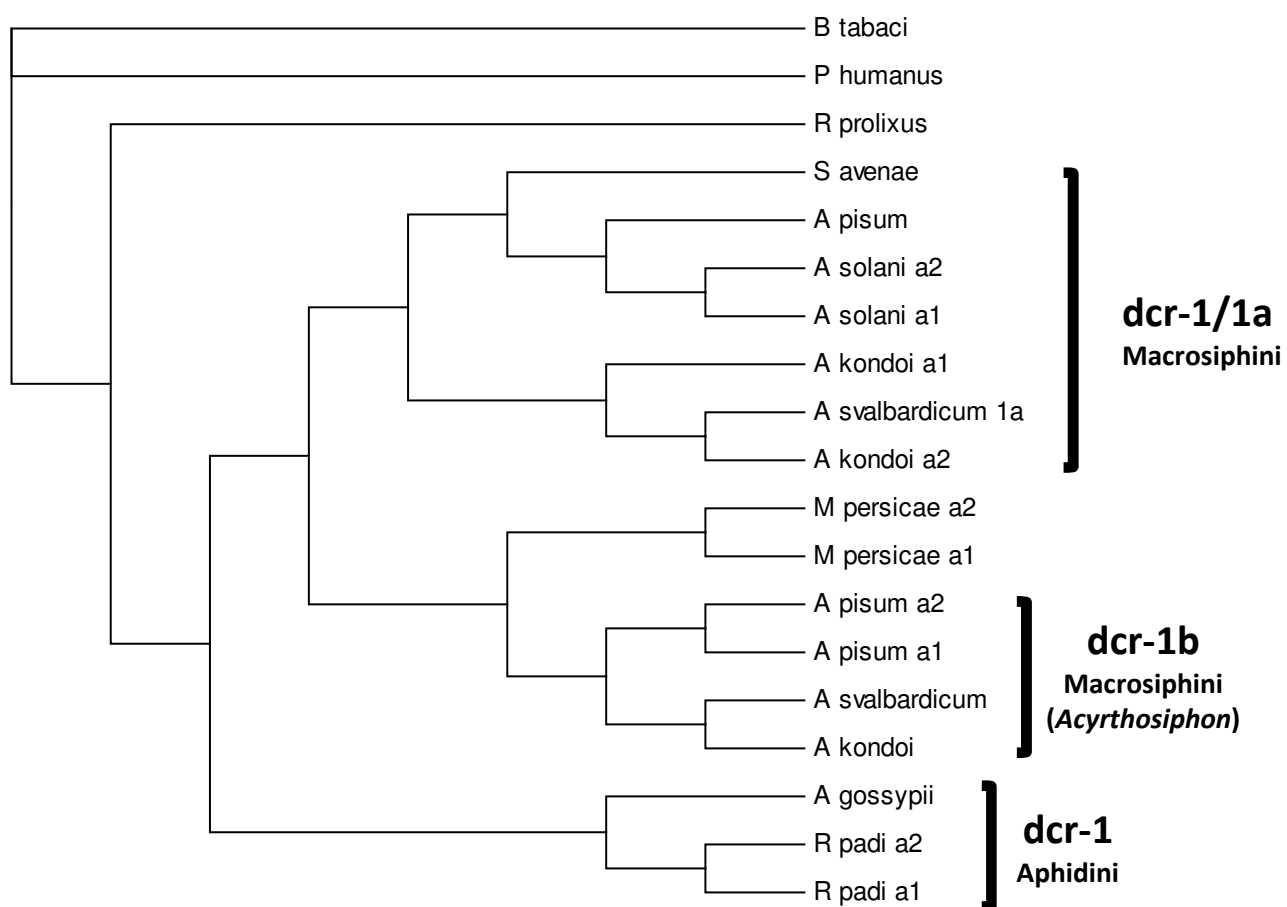

## Hypothesis 7

Supplement: Additional file 1 — Figure S3. Test of alternative hypotheses for the phylogeny of dcr-1 in aphids. [file 1471-2148-12-216-S1.pdf]
