# Supplementary material for: Evolutionary study of duplications of the miRNA machinery in aphids associated with striking rate acceleration and changes in expression profiles
Source: BMC Evol Biol. 2012 Nov 12;12:216. doi: 10.1186/1471-2148-12-216 (PMC3536612; doi:10.1186/1471-2148-12-216)
Supplement: Additional file 2 — Figure S1. Further duplication of dcr-1 in Acyrthosiphon kondoi and Acyrthosiphon svalbardicum. [file 1471-2148-12-216-S2.pdf]

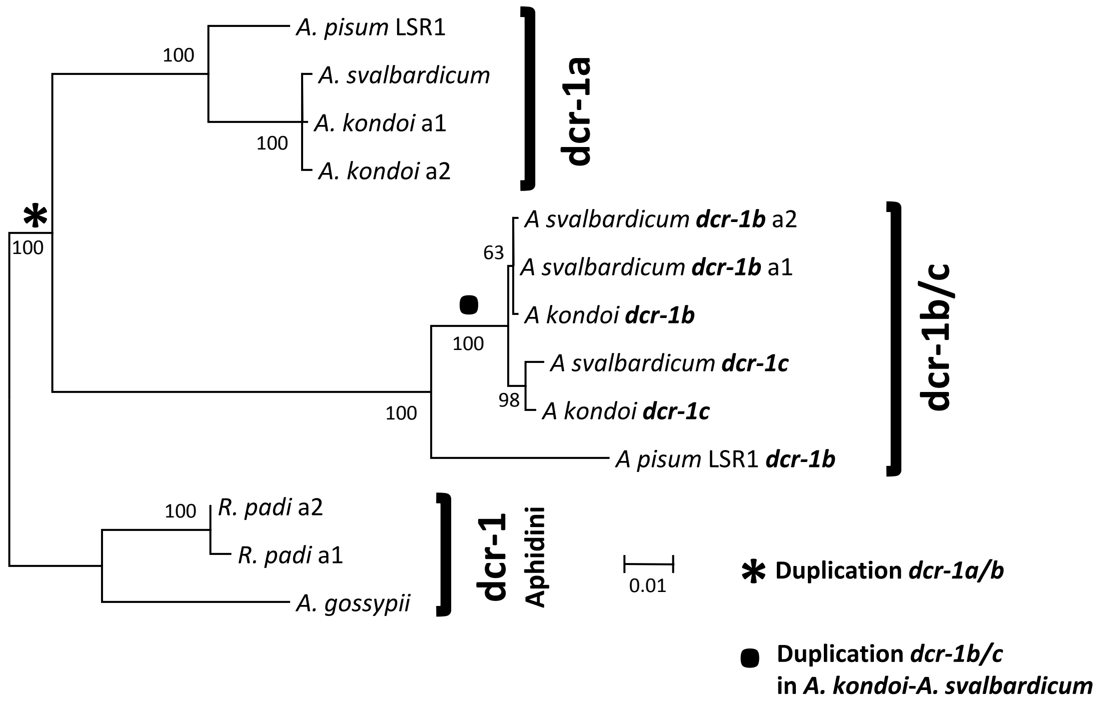

Supplementary Figure 1. Further duplication of *dcr-1* in *Acyrthosiphon kondoi* and *Acyrthosiphon svalbardicum*. The amplification of a longer fragment of ~2000 nucleotides in these two species allowed the sequencing of a second allele of *dcr-1b* in *A. svalbardicum*, proving that the *dcr-1c* sequences are indeed a new copy of the gene and not divergent alleles of *dcr-1b*. The maximum likelihood reconstruction (model HKY+G) shows a very close relationship between the *dcr-1b* and *dcr-1c* copies of *A. kondoi* and *A. svalbardicum*, which suggests that the new duplication arose from the *dcr-1b* copy and that it is independent from the other new duplication found in *A. pisum*. An asterisk (*) marks the suggested moment of the duplication of *dcr-1a* and *dcr-1b* and a square (■) marks the suggested moment of the duplication of *dcr-1b* and *dcr-1c*.
