# Supplementary material for: Evolutionary study of duplications of the miRNA machinery in aphids associated with striking rate acceleration and changes in expression profiles
Source: BMC Evol Biol. 2012 Nov 12;12:216. doi: 10.1186/1471-2148-12-216 (PMC3536612; doi:10.1186/1471-2148-12-216)
Supplement: Additional file 3 — Figure S2. Further duplication of dcr-1 in Acyrthosiphon pisumLSR1. [file 1471-2148-12-216-S3.pdf]

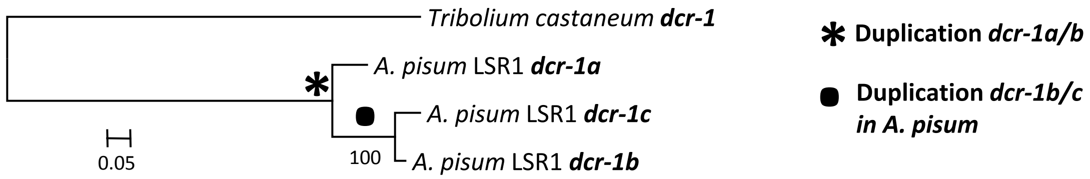

Supplementary Figure 2. Further duplication of *dcr-1* in *Acyrthosiphon pisum* LSR1. The maximum likelihood reconstruction from a 615 amino acid alignment (model JTT+I+F) shows that the duplication arose from the *dcr-1b* copy. An asterisk (*) marks the suggested moment of the duplication of *dcr-1a* and *dcr-1b* and a square (■) marks the suggested moment of the duplication of *dcr-1b* and *dcr-1c*. The sequence of *dcr-1c* of *A. pisum* LSR1 was not included in the phylogeny of Supplementary Figure 1 due to total lack of homologous positions.
