# Supplementary material for: Evolutionary study of duplications of the miRNA machinery in aphids associated with striking rate acceleration and changes in expression profiles
Source: BMC Evol Biol. 2012 Nov 12;12:216. doi: 10.1186/1471-2148-12-216 (PMC3536612; doi:10.1186/1471-2148-12-216)
Supplement: Additional file 4 — Table S1. PCR primers used in this study, ordered in relative positions from 5’ to 3’ of the corresponding gene/region. [file 1471-2148-12-216-S4.pdf]

Supplementary Table 1. PCR primers used in this study, ordered in relative positions from 5' to 3' of the corresponding gene/region.

Primer name	Primer sequence (5' to 3')	Forward (f) or reverse (r)	Notes
ago-1			
Region 1			
Ago1Reg1F1	ccaaatytrggacgagaa	f	
Ago1a-r1F1	ccgaccaattgtgcttcgtg	f	Specific to copy -1a
Ago1b-r1F1	ccgaccaattaggcttcgta	f	Specific to copy -1b
Ago1a-r1F2	ccattgcctattggaaacga	f	Specific to copy -1a
Ago1b-r1F2	ccattgcctattggaaggaa	f	Specific to copy -1b
Ago1b-r1F3Mp	tgggtgaatcattagaaggtca	f	Specific to copy -1b of *M. persicae*
Ago1b-r1F3	ggcwgcagaagaccaatacc	f	Specific to copy -1b
Ago1b-r1R2	tagatggctgcawtgactgg	r	Specific to copy -1b
Ago1b-r1R2Mp	tagatggccggagtgactgg	r	Specific to copy -1b of *M. persicae*
Ago1Reg1F2	ccwwcwmaatggaaaatgatgct	f	
Ago1Reg1R1	ttgcrtttgagctggtyttc	r	
Ago1a-r1R1	tgcttacgattgcatttgag	r	Specific to copy -1a
Ago1b-r1R1	tgcttacgtttgcgtttgag	r	Specific to copy -1b
Region 2			
Ago1Reg2F1	aaggmaactgyaagragtg	f	
Ago1a-r2F2	ctcctgatcgtgaacgagag	f	Specific to copy -1a
Ago1b-r2F2	ctcctgcacgtgaacaagat	f	Specific to copy -1b
Ago1Reg2F2	caatagcttgytttgcayca	f	
Ago1a-r2F1	gtgcgtgaggatgcgttaag	f	Specific to copy -1a
Ago1b-r2F1	gtgcgcgatgatgctataaa	f	Specific to copy -1b
Ago1Reg2F3	kcyacwcaatgtgttcaagc	f	
Ago1Reg2F4	caatgtgttcaagcdaaaaat	f	
Ago1Reg2R2	attgrtaccrcccaatttca	r	
Ago1b-r2R1Ag	gcagcaattgattgattgttaatg	r	Specific to copy -1b of *Aphis gossypii*

<u>Ago1Reg2R1</u>	ccttcgcatactccatcrcg	r	
<u>Ago1a-r2R1</u>	actgtccttcggatactcca	r	Specific to copy -1a
<u>Ago1b-r2R1</u>	gctgtccttcggatactcca	r	Specific to copy -1b
Region 3			
<u>Ago1Reg3F1</u>	agckattcgagaggcaatgt	f	
<u>Ago1a-r3F1</u>	cagctattcgagaggcatgt	f	Specific to copy -1a
<u>Ago1b-r3F1</u>	tggcgattcgagaggcatgt	f	Specific to copy -1b
Ago1b-r3R2Ag	ccgggtttgtaatccacttc	r	Specific to copy -1b of *Aphis gossypii*
Ago1a-r3R2Rp	ttccggattgttcctttttg	r	Specific to copy -1a of *Rhopalosiphum padi*
Ago1b-r3R2Rp	attcatccgactcgaaatgg	r	Specific to copy -1b of *Rhopalosiphum padi*
Ago1a-r3R2Ag	atgacttgggcgacttgttc	r	Specific to copy -1a of *Aphis gossypii*
<u>Ago1a-r3R1</u>	ccggtgcaggtatagacaca	r	Specific to copy -1a
<u>Ago1b-r3R1</u>	ccggtgcaggtatagatata	r	Specific to copy -1b
<u>Ago1Reg3R1</u>	ccggtgcaggtatagayaya	r	
dcr-1			
Dcr1bF0	cctaattggatggagataggg	f	For longer sequence in *Acyrthosiphon* spp
<u>Dic1abF1</u>	tgggarttaaattcaaacactgg	f	
Dcr1abF2	tccrggrccaartccwagt	f	
Dcr1abF3	ttrgaaacrathggwgattc	f	
Dcr1bF2	ttgagccccgtagtaattgg	f	For longer sequence in *Acyrthosiphon* spp
Dic1abR2	ggcatgrgtcatdgcytgta	r	
Dcr1bR2	aatgcaccacgantaccaca	r	For longer sequence in *Acyrthosiphon* spp
<u>Dic1abR1</u>	cgattgggrtaataagaagca	r	
Dcr1bR0	ccattctcttcttggatagc	r	For longer sequence in *Acyrthosiphon* spp
Dcr1bR3	cccgcaactgattcaaaaat	r	For longer sequence in *Acyrthosiphon* spp

The primers mainly used for amplification of each region/gene are <u>underlined</u>.
