# Supplementary material for: Evolutionary study of duplications of the miRNA machinery in aphids associated with striking rate acceleration and changes in expression profiles
Source: BMC Evol Biol. 2012 Nov 12;12:216. doi: 10.1186/1471-2148-12-216 (PMC3536612; doi:10.1186/1471-2148-12-216)
Supplement: Additional file 5 — Table S2. PCR primers used for semiquantitative RT-PCRs. [file 1471-2148-12-216-S5.pdf]

Supplementary Table 2. PCR primers used for semiquantitative RT-PCRs

**Gene**	**Primer**	**Sequence (5'-3')**	**Tm**	**cycles**
Api-dcr1a	Api-dcr1aF	TGGGAGTTAAATTCAAACACTGG	60	38
	Api-dcr1aR	CGATTGGGGTAATAAGAAGCA	60	38
Api-dcr1b	Api-dcr1bF	CAGCAGCCAAATGTGCTTTA	56	38
	Api-dcr1bR	CAATTCACTCTGATCAATCTATTCAAA	56	38
Api-ago1a	Api-ago1aF	AAAAAGATTACTGCGCAACAT	58	36
	Api-ago1aR	TGTTTGTTACAACTTAACCCC	58	36
Api-ago1b	Api-ago1bF	ATTGGCGCGAGCTATTACAG	58	38
	Api-ago1bR	CACGAAATTTACACGGAAACG	58	38
